# A Manual of Surgical Treatment

**Published:** 1913-03

**Authors:** 


**A Manual of Surgical Treatment.** By Sir W. Watson Cheyne Bart., C.B., and F. F. Burghard, M.S., F.R.C.S., with the assistance of T. P. Legg, M.S., F.R.C.S. and Arthur Edmunds, M.S., F.R.C.S. New Edition entirely revised and largely rewritten. Volume III. Pp. xxviii, 575. London: Longmans, Green & Co. 21s. net.

This volume deals with the injuries and diseases of joints and spine and the surgical affections of the head and neck. The section on the injuries of joints contains but little fresh matter, but the operative treatment of complicated dislocations is brought up to date. The chapters dealing with tuberculosis are of that high order of merit which we should expect from one who has devoted so much attention to the subject. They are of the greatest value in containing so many and such precise details for the application of treatment to every variety of this multiform disease. Gauvain's various methods of conservative treatment of tuberculous spine are given in some detail, indicating the great importance attached by the authors to this aspect of the subject.

The chapter on cranial injuries is written on the usual lines of English text-books. The question of decompressive operations for cases of fractured base with prolonged coma is not dealt with, nor is that of intra-cranial hemorrhage in the newly born.

The modern technic for operations on cerebral tumours is well described, but here again there is a disappointing blank, in the subject of pituitary tumours being omitted altogether.

The chapter on trigeminal neuralgia is little more than an account of various operative details, without much guidance being given as to the comparative merits of each method. No opinion is expressed as to the value of the alcohol injection method, and the possibility of dividing the sensory root of the Gasserian ganglion without removal of the latter is not mentioned.

